# Branding the Earth: Selling Earth system science in the United States, 1983-1988

**DOI:** 10.1177/03063127221122436

**Published:** 2022-09-12

**Authors:** Jenifer Barton

**Affiliations:** University of Toronto, Toronto, ON, Canada

**Keywords:** NASA, Earth sciences, Earth system science, branding

## Abstract

As part of its efforts to find new relevance in the early 1980s, NASA formed the Earth System Sciences Committee (ESSC) to develop a large-scale Earth science research program that would use satellites and computer modeling to study the planet as an integrated system with interconnections between the land, air, water, and biota. Called Earth system science (ESS), the project was conceived on the scale of the U.S. moon missions. Like the Apollo program, it would need enormous government funding to implement. Yet, the project was proposed just as government science funding was contracting. Conscious of the changing political economy of science, the ESSC attempted to build scientific, political, and public support for its project by using promotional techniques akin to the branding efforts more commonly identified in corporate marketing that were themselves changing in scope and importance in the 1980s. These techniques formed part of the ESSC’s broader management strategy to promote ESS. The ESS brand was developed around the ideals of an interconnected ‘Earth system’, the significance of interdisciplinary research, and environmental concern. Though ESS failed to gain widespread traction, an unintended consequence of this branding was the communication and entrenchment of the concept of the ‘Earth system’. Today, this concept provides crucial theoretical scaffolding that unifies interdisciplinary Earth science research, including climate change science.

In 1984, Nike and NBA rookie Michael Jordan entered into a mutually transformative relationship. Using highly stylized television commercials, advertisements and a newly created line of Air Jordan sneakers, Nike linked its corporate identity to the athleticism of Michael Jordan. The relationship revolutionized Nike. Before Jordan, it was a company that sold material products, specifically running shoes. After Jordan, Nike’s brand stood for something more abstract. It signified sports, fitness and the veneration of athletes (Klein, 1999, pp. 50–53). Similarly, during the 1984 Super Bowl, Apple aired its now-famous ‘1984’ commercial for the new Macintosh personal computer.^1^ Directed by Ridley Scott, the commercial was set in a dystopian future where mindless workers sit transfixed by an authoritarian figure looming over them on a large screen. A lone female runner enters the room and with the sweep of a sledgehammer destroys the screen, presumably liberating the workers. The commercial ended by telling viewers that they’ll ‘see why 1984 won’t be like *1984’*. The unsubtle suggestion was that to buy a Macintosh was also to buy the values of Apple’s brand – individualism, autonomy and creativity – while simultaneously rejecting the ‘Big Brother’ values of Apple’s main competitor, IBM. These corporate branding campaigns are now familiar illustrations of the way branding was transformed in the 1980s, a transformation that also had effects in the sciences, as this paper will argue.

With the election of Ronald Reagan and Margaret Thatcher in the U.S. and U.K. respectively, those governments returned to pursuing policies that privileged private enterprise. These policies, among other things, contributed to intensifications of a global trade regime that enabled particular transnational flows of capital and goods. As a consequence, manufacturing declined in Western nations, as corporations increased profit margins by moving production to what were then euphemistically called low-cost geographies (Harvey, 2005; Mirowski, 2011, pp. 25–31). What economists like Joseph Stiglitz critically describe as ‘globalization’ presented many for-profit corporations with an odd paradox (Stiglitz, 2002). On the one hand, a global market meant there were opportunities for enhanced profit margins, new sales and new products. On the other hand, a global market also meant that it was harder for products to stand out among competitors. Further, if production and other corporate activities were increasingly outsourced (in whole or part), the reason for having a corporation was unclear. In short, in the 1980s, there was a heightened sense of competition and a modest crisis of corporate identity (Moor, 2007).

In response, many corporations turned to new ways of branding to build distinct and coherent identities. Of course, branding has been used for centuries to mark ownership or contents. However, it was only in the late nineteenth and early twentieth centuries – a period sometimes referred to as the Second Industrial Revolution – that what Williams (1980) calls the ‘magic system’ emerged. In an effort to make products stand out among a multitude of nearly identical competitors, companies began using packaging and advertisements to build brands that associated their products with qualities and virtues – for instance, ‘purity’ and ‘trustworthiness’, in the case of Quaker Oats – that would differentiate them from competitors’ products (Lury, 2004, p. 75; Moor, 2007, pp. 16–19; Williams, 1980, pp. 170–195). In the 1980s, branding was even further transformed when globalization detached corporate production from specific locations and many corporations outsourced production altogether. As corporations began to brand their own identities rather than individual products, brands became more abstract. Brands became linked less to specific material objects and more to intangible values, beliefs, meanings, and lifestyles. A brand incorporated a company’s products, but it was not reducible to them (Arvidsson, 2006, pp. 2–3; Moor, 2007, pp. 33–35). Along with Nike and Apple, entities like Disney, Levi, Starbucks and Coke all developed new corporate imaginaries – their ‘brands’ – through meticulously produced television commercials, celebrity endorsements, culture and sports sponsorships, and the opening of brand stores. Consumers now bought brands, not products. The consumption of brands offered more than the narrow satisfaction of material wants. In principle, brands provided unique ‘experiences’ that created opportunities for individuals to purchase their own identities.

When brand culture was transformed in the 1980s, it was also embraced by non-commercial groups and used to achieve goals other than simply increased profits. Charity and activist organizations such as UNICEF and the World Wildlife Fund rebranded in the 1980s and later (Moor, 2007, pp. 4–5), and Britain’s Labour Party became ‘New Labour’ in the mid-1990s. Although non-commercial branding always has a financial component (for instance, increasing donations), the ultimate goal and aim of these groups (unlike, say, Nike and Apple) is arguably something other than profit for the sake of profit (such as alleviating child poverty or wildlife protection). The key issue here, as Moor (2007) points out, is that it is a mistake to think of commercialization as the defining feature of brands. Branding, in its broadest form, is a *management* technique. As Moor puts it, branding is ‘the effort to pattern information – and to embed informational qualities in material goods – in order to organize experiences and perception in line with particular strategic ends’ (Moor, 2007, p. 9). This feature of branding is shared by commercial and non-commercial branding activities alike.

This article focuses on another example of non-commercial branding, a case study in the Earth sciences where techniques that we could now label as ‘branding’ were used as part of a broader management strategy to promote a major research program. In the mid-1980s, a group of scientists and administrators working within the institutional structures and mechanisms of the National Aeronautics and Space Administration (NASA) used branding techniques to create a community of like-minded supporters. The group was the Earth System Sciences Committee (ESSC), and its ambitious aim was to create a research program called Earth system science (ESS) that would integrate researchers from across the Earth sciences. ESS was proposed as an interdisciplinary endeavour that would study the whole Earth in terms of interconnections between the air, land, water and biota. To make this research possible, the project required a costly material infrastructure: a network of Earth-observing satellites for data collection and supercomputers to store data and run numerical models. This infrastructure would need extensive government support that, in turn, required public engagement. To give the project its best chance of being funded, the ESSC deployed a strategy that incorporated many of the branding techniques that became increasingly popular in the 1980s to promote the ideals of an interconnected Earth – the ‘Earth system’ – along with the value of interdisciplinary research. While those around the ESSC variously described this work as a ‘promotional strategy’, a ‘trademark’ and ‘selling’, the recurring use of specific visual cues and thematic elements may be described as science branding. This description does not impugn the ESSC’s work, but acknowledges the political economy of science in which the committee operated.

What makes this case particularly interesting is that the branding of ESS was not limited to familiar strategies such as the use of logos, colour schemes and letterheads (though these techniques were used), but also permeated the scientific content of ESS. As will be seen, the project’s initial data collection and modeling goals were outlined in a technical ‘wiring diagram’ of the ‘Earth system’ that conceived the entire Earth as a system made up of interconnected subsystems. A simplified version of this wiring diagram – what came to be called the Bretherton diagram – was designed specifically to show Earth scientists exactly where their disciplines fit into the overall interdisciplinary project. The Bretherton diagram was used as a management tool by the ESSC to promote the ESS values of interdisciplinarity and an interconnected planet. In the cultural context of the 1980s, the Bretherton diagram is best understood as part of the broader branding strategy to promote ESS to divergent communities.

Despite its efforts, the ESSC ultimately failed to achieve its principal objective. Its research program received only lukewarm responses from some Earth scientists rather than wholesale, unanimous support. The immense cost of ESS potentially jeopardized research in other domains by threatening to vacuum up increasingly scarce government research dollars. The project’s prioritization of space-based observations and shorter timescales also created ‘winners’ and ‘losers’ among Earth science disciplines (Conway, 2008). However, I argue that the ESSC’s extensive promotional work paid off in a way that was both unintended and unexpected. It made a significant contribution to the early entrenchment of the ‘Earth system’ concept that broadly captures the idea that the planet is an integrated system comprised of interconnected components in the air, land, water and biota. Nascent sketches of this idea had been growing among Earth scientists since the end of World War II under many guises – Gaia and Spaceship Earth, to name just two (Dutreuil, 2020; Höhler, 2015) – but they were poorly articulated and none had become widely endorsed in scientific communities (Barton, 2020).^2^ In contrast, immediately after the ESSC launched its major promotional campaign in 1986, the ‘Earth system’ phrase began to proliferate in the Earth sciences and elsewhere. Those involved in developing and promoting ESS may not have explicitly described their activities as ‘branding’. Nevertheless, many of the ESSC’s activities have a striking family resemblance with the prevalent brand development techniques that emerged during the 1980s. I argue that the ESSC’s extensive promotional efforts are best described as branding and contributed to the spread and later entrenchment of the ‘Earth system’ concept. I further suggest that the new ubiquity of the phrase ‘Earth system’ signals an ontological shift, whereby the Earth became more widely recognized as a new kind of thing. Today, among Earth scientists, the Earth is no longer merely *studied* as a system, the Earth *is* a system.

This case study shows how the branding strategies of the 1980s arguably shaped the management of the Earth sciences. The ESSC branded the whole Earth as the ‘Earth system’, marking the planet as an interconnected system amenable to interdisciplinary scientific study. Though it used many promotional techniques akin to those used in commercial and organizational branding efforts that were increasingly popular in the 1980s, the ESSC was not able to counteract the trend of shrinking government funding for science and technology during the latter years of the Cold War, nor was the group able to achieve its primary goal of securing support for a largescale Earth science research program. Nevertheless, these branding techniques had a lasting effect in the Earth sciences by contributing to the early spread and entrenchment of the concept of the ‘Earth system’. Branding techniques helped remould the Earth into what Daston (2000) calls a ‘scientific object’, an object amenable to a scientific study. This concretized a new scientific understanding of the planet, an understanding that now undergirds much contemporary research, including climate change science.

## Sell, sell, sell

NASA was founded, in large part, to produce space spectacles to compete with the Soviets. From its inception in 1958 through the Mercury and Gemini programs to the last Apollo mission in 1972, NASA’s unstated aim was to publicly demonstrate the scientific and engineering virtuosity of the U.S. By proxy, the goal was to demonstrate the superiority of capitalist liberal democracy over communist centralized planning. The so-called ‘space race’ arguably ended with the 1969 moon landing. In the early 1970s, competition with the Soviets was so diminished that American astronauts and Soviet cosmonauts would ‘shake hands’ in space as part of the Apollo-Soyuz Test Project in 1975. The waning of Cold War tensions made costly prestige projects less attractive to the U.S. Congress. By the mid-1970s, this was reflected in the contraction of government funding for scientific research as Congress began to favour more practical research (Wolfe, 2013).

In the changed geopolitical climate, NASA needed a new raison d’être (Kay, 2005). NASA’s response was to turn ‘back to Earth’, putting a new focus on its capacity to build and launch Earth-observing satellites for Earth science research (Conway, 2008, 2014; Maher, 2017). This earthward turn began with relatively small-scale and targeted research on ozone depletion in the 1970s (Lambright, 2005). There were, however, ambitions at NASA to get Congressional support for a much larger Earth science research program. An early attempt to formulate just such a program was ‘Global habitability’, which received a startlingly hostile reception at an international space conference in 1982, largely because it lacked a comprehensive data collection and access plan (Barton, 2021; Conway, 2008, pp. 219–225). Scarred by this experience, but undeterred, in 1983 NASA officials formed the ESSC to put together a scientific rationale for a large-scale program to study the Earth as an interconnected system using satellites, computer models and complementary ‘in situ’ ground measurements. The motivation for such a program was bolstered by the growing awareness of environmental problems – notably ozone depletion, acid rain, eutrophication of waterways, deforestation, desertification and the ‘Greenhouse effect’ – all caused by human activities (NASA Advisory Committee [NAC], 1988, p. 76). It was also supported by a growing belief in the Earth sciences that, with new technologies like Earth-observing satellites and digital computer models, the Earth could be scientifically studied in more comprehensive and interconnected ways (Edwards, 2000, 2010).

The ESSC did not represent a particularly new or innovative approach to scientific management. It joined the tediously long list of post-World War II advisory committees, such as those of the National Academy of Sciences (NAS), formed to provide scientific and technical guidance, often for interdisciplinary and large-scale projects that would require extensive government funding. Science committees like the ESSC had become common cogs in the machinery of scientific bureaucracy in the U.S. as government funding for science expanded during the Cold War. The use of such committees continued even as government funding began contracting in the 1970s and continued to contract in the 1980s (Mirowski, 2011; Wolfe, 2013). When formed, the ESSC had 15 members made up of scientists and engineers from university, government and industry, representing a cross section of Earth science disciplines: atmospheric physics and chemistry, physical and biological oceanography, ecology, geophysics, geology, cryology and solar-terrestrial physics.^3^ Francis Bretherton, an applied mathematician who worked on ocean models, was appointed chair. A further 239 scientists and administrative support staff would contribute to the preparation of the ESSC’s reports, either through participation in one of the committee’s working groups or by reviewing reports before publication (NAC, 1988, pp. 202–203).

The committee represented diverse disciplinary interests because its ambition for the Earth sciences was not modest. The goal was to initiate a major Earth science research program that would integrate the various disparate disciplines using a ‘systems’ approach to study the Earth as if it were a system of interconnected systems. The atmosphere, hydrosphere, cryosphere, geosphere and biosphere were all construed as ‘subsystems’ of the overarching ‘Earth system’. This view of the Earth now may be familiar, but it is commonplace in no small part because of the ESSC’s work, outlined in its two published reports: *Overview* (1986) and *A Closer view* (1988). Given its grand design, it was likely overdetermined that the ESSC recommended a comprehensive and expensive research program. ESS was envisioned as a global Earth science research program that would work towards building a more comprehensive understanding of Earth processes and their interactions. These processes ranged over various timescales. There were short-term processes that might last only seconds, days, weeks or years (e.g. atmospheric turbulence, atmospheric convection, volcanic eruptions, earthquakes, seasonal vegetation changes, global weather systems). Then, there were medium-term processes that occurred on timescales of decades to centuries (e.g. soil erosion, climatic conditions, carbon dioxide variations, ocean circulation, nutrient cycles). Finally, there were long-term processes that occurred on timescales of thousands to millions of years (e.g. glacial periods, soil development, speciation, atmospheric composition, plate tectonics, mantle convection, mountain formation) (NAC, 1988, p. 27).

The overarching ‘goal’ of ESS was: ‘To obtain a scientific understanding of the entire Earth system on a global scale by describing how its component parts and their interactions have evolved, how they function, and how they may be expected to continue to evolve on all timescales’. The ESS would be global in scope – focusing on changes observable on a regional or planetary scale, rather than smaller spatial scales – but, given the immensity of the task, not all timescales could be given equal priority. Therefore, the ESSC set a more achievable ‘challenge’ for ESS that focused on the timescale most relevant to human lifespans, decades to centuries. ESS’s narrower ‘challenge’ was: ‘To develop the capability to predict those changes that will occur in the next decade to century, both naturally and in response to human activity’. Meeting the ‘challenge’ of predicting changes on medium-term timescales of decades to centuries was of ‘great urgency’, given the many environmental problems facing the planet (NAC, 1988, p. 11). The technological centerpiece of the program was to be a fleet of Earth-observing satellites, together forming the Earth Observing System (EOS), able to collect simultaneous data on a variety of key global environmental variables. Numerical models running on powerful supercomputers would calculate past and present Earth system conditions and (hopefully) make accurate predictions about future global trends (NAC, 1988, pp. 134–171). A massive information system would be needed to facilitate data reduction, analysis and numerical modeling, along with more mundane, yet equally crucial, capabilities for data storage and access (NAC, 1988, p. 31).

Nothing like this – on such a global scale, with this depth of interdisciplinarity, with this much reliance on Earth-observing satellites and numerical modeling – had ever been proposed before in the Earth sciences. This was a project with a scale and ambition commensurate with the Apollo program. And like Apollo before it, ESS would not be cheap. ESSC members and affiliated experts were acutely aware that, in order to make ESS possible, they would need to convince many different constituencies that it was worth the time and the resources. Of course, the project required a widespread scientific and engineering consensus among Earth scientists, satellite engineers and computer scientists to bring scientific aims into step with available satellite instrumentation and computational capacity. But, more importantly, given that the project would need extensive government funding, the whole bureaucracy of U.S. scientific management would need to be mobilized. This included the civil servants and political appointees who directed the alphabet-soup of U.S. science agencies and departments (NAC, 1988, p. 161).^4^ This also included Congress, which holds tight the government’s purse strings. Getting Congress on board would require making connections with Congressional staffers who were forever being courted through formal channels and informal backroom deals. Allies would need to be enlisted among the politicians sitting on the Congressional committees responsible for authorizing and appropriating the government’s discretionary spending. Then there was ‘the public’ to consider. Public support could translate into political support that might turn into financial support. For the ESSC to develop its research program, many groups had to be reached persuasively, not just scientists and engineers.

The ESSC produced a ‘Working framework’ (1984) that candidly assessed the group’s undertaking: ‘The task before the Committee is daunting, requiring the establishment and effective presentation of a consensus of many diverse interests, as well as a realistic resolution of many technical and program issues within a total level of effort that is severely constrained by budgetary and institutional factors.’ This meant that, ‘The conclusions of the ESSC effort must also be clearly communicated in a timely and persuasive manner.’^5^ Committee members acknowledged the communication challenge, noting that ‘advocacy’ would be key to their success. As one member put it, the ESSC must ‘sell, sell, sell’, using language evocative of David Mamet’s contemporary *Glengarry Glen Ross*.^6^ To achieve its goals, the ESSC needed to make their reports not only scientifically sound and accessible, but also exciting and visually appealing. That was no easy task. Since Thomas Sprat and Robert Boyle in the seventeenth-century Royal Society, scientists and engineers had been trained to write in plain and dry prose, what Shapin (1984, p. 495) describes as a ‘naked way of writing’. The transition to persuasive prose and effective rhetoric was not easy.

Early ESSC draft documents very often fell short of rhetorical expectations. When this happened, ESSC members were quick to point out the shortcomings. John Dutton, ESSC member and atmospheric physicist, thought that the ESSC’s early ‘Working framework’ was an appallingly dull document that failed to generate excitement or to distinguish itself.^7^ It was so ‘bland’ that he described it as a ‘dangerous document’. The report might shape many first impressions of ESS, and if it failed to generate excitement it could kill the project before it even started. Dutton’s worry was that, ‘If this report gives the impression that we are just muddling around and don’t have a focus, then our entire effort will be compromised.’ Dutton thought the ESSC needed to be clear about why the ‘Earth System view is essential’ and why this view was, ‘worth half a billion a year of the taxpayers’ money’. In his opinion, the draft ‘Working framework’ failed on both accounts. Dutton insisted that in its present form it was ‘not really very much to hang a hat on. I would think we would want to go to the world with something that generates more excitement and is somewhat crisper.’ He recommended having ‘a stirring wrap-up, something to salute, before we trundle off to the details of working groups, members, etc.’ As it stood, ‘The document itself is not nearly ready to go public.’^8^

Jack Eddy, a solar astronomer, reviewed a draft of the ESSC’s *Overview* report. He described the document as ‘an unusual mixture of TV salesmanship (at the start), powerful science statements (in the middle), and all-too-terse and telegraphic budget curves and tables (at the end).’ Eddy claimed that the first part of the report ‘reads like a revival meeting sermon’ or an ‘advertising brochure’ that was written in the ‘hackneyed style of a NAT’L GEOGRAPHIC article’. He compared some of the early ‘salesmanship’ phrasing to ‘a mayor’s proclamation of Guinea Pig Week’. Eddy meant these comments to be taken as constructive criticism, not as an indictment of the overall project. He assured ESSC members: ‘I hope also that any who read this know that it comes from one of the [ESS] Apostles, an admirer of the accomplishments of the ESSC, and one who may not fully appreciate the need for popularizing the study at this time.’ Though critical of the ESS report’s variable tone, Eddy recognized the need to ‘sell’ the program and recommended simplifying the writing. He commented: ‘In places it reads like a NASA engineering-bureaucratese document.’^9^ For anyone not familiar with this genre of writing, Eddy’s comment was *not* a compliment. Eddy’s point was that, if the ESSC planned to persuade civil servants, politicians and the public, then they needed a consistent and engaging style. They could not afford to simply flit back and forth between sales pitches and arid technical writing.

## Payson Stevens and InterNetwork

Members of the ESSC recognized that they needed to use persuasion to build a broad consensus beyond technical specialists in the Earth sciences, satellite engineering and computational modeling. They further recognized that the style of technical writing that lent itself to convincing scientists and engineers would not compel bureaucrats of science, politicians or the public. Recognizing the need to ‘sell, sell, sell’, however, did not translate straightforwardly into a capacity to pitch science in a way that would engage crucial non-technical constituencies. Confronted with this communication problem, in late 1985, the ESSC turned for help to an expert in graphic design and science communication: Payson Stevens and his company InterNetwork Inc (INI). Stevens and INI prepared all ESS material and a promotional strategy more broadly, making use of the techniques more often associated with corporate marketing, but that were increasing in scope and influence in the 1980s. With a background in molecular biology and biological oceanography as well as graphic design, Stevens had experience preparing media presentations of scientific findings. After completing some graduate work at the Scripps Institution of Oceanography under the guidance of Roger Revelle, Stevens moved permanently into multimedia science communication. Commenting on Stevens’ subsequent work, Revelle acknowledged that Stevens was ‘a pioneer in this field of combining science and art and I’m enthusiastic about it’.^10^

ESSC Chair Francis Bretherton recognized that Stevens played a crucial role in developing the *Overview* report:

He [Stevens] made major contributions in clarifying the basic approach toward communicating with a broad audience. The design of the *Overview* both in concept and execution were his responsibility. To do this, he had to work effectively with a broad community of scientists. His energy and enthusiasm were infectious … and I appreciated the opportunity to work with him.^11^

Stevens was described as having ‘a flashy character’ and being full of enthusiasm for ‘big picture’ ideas.^12^ Nancy Ann Brewster from the National Science Foundation (NSF) wrote to ESSC manager Laura Lee McCauley that, ‘I loved meeting Payson, what a character, and must be fun to work with’. McCauley wrote back: ‘Yes, Payson is a kick to work with.’^13^ ESSC member and ecological modeler Berrien Moore described Stevens as a ‘very creative person … [who] had the ability to pull all of this material together with very good graphical imagery and it [the ESSC report] became a really monumental document’ because of Stevens’s work (Moore, 2011).

Stevens formed his company InterNetwork in 1981 to provide communication services to organizations wanting to convey scientific information to broader, lay audiences using a variety of media.^14^ By the late 1980s, his reputation was such that he was invited to make a presentation at the Sundance Symposium on Global Climate Change held in August, 1989. The conference brought together over 200 scientists and other interested parties from the U.S. and U.S.S.R. to discuss climate change. The symposium was nicknamed ‘Greenhouse Glasnost’ in a nod to the reforms for political transparency enacted in the Soviet Union under Mikhail Gorbachev. Funded in part by the actor Robert Redford’s Institute for Resource Management, Stevens attended the conference alongside a high-profile group of participants that included Redford, James Lovelock, James Hansen, Carl Sagan and Paul Ehrlich (Minger, 1990). After the conference, Redford wrote to Stevens with words of praise: ‘The creative energy and imagination you invested was obvious. It was a tremendous demonstration of putting high technology to its highest possible use – combining art and computers to educate influential decision-makers about one of the most serious issues facing humanity.’^15^ In April and May of 1990, Senator Al Gore chaired the Interparliamentary Conference on the Global Environment that brought together experts and politicians to discuss the many environmental challenges facing the planet. The proceedings included a keynote address by Carl Sagan and a ‘hypermedia presentation’ by Stevens.^16^ INI would go on to receive the Presidential Award for Design Excellence from Bill Clinton in 1994.^17^

The roots of Stevens’ association with NASA trace back to 1979, when the director of NASA’s Earth Observations Program hired physical oceanographer W. Stanley Wilson. Wilson was tasked with creating a comprehensive ocean remote sensing program to follow up on the successful but short-lived. Seasat satellite launched on 27 June 1978 (Conway, 2006, p. 140).^18^ Part of Wilson’s strategy to build the program was to promote the use of satellite data. Expanding the community of satellite data users could effectively increase the demand for satellite data and, eventually, translate into demand for new satellites. The problem was that already available satellite data was being barely used. Part of the problem may have been that, until the mid-1970s, satellite data were presented in tables and graphs with little attempt to arrange them in accessible or compelling ways. This meant that, for experts unfamiliar with interpreting satellite data, it took considerable effort to reveal underlying patterns. The data was not able to grab attention. Making the data accessible to a larger scientific community required a way of depicting information that revealed patterns that were not readily apparent in tables or graphs.

The need to present satellite data in visually compelling ways to generate widespread interest and use was exacerbated after NASA launched ERTS-1 (Earth Resources Technology Satellite-1, renamed Landsat 1 in 1975) on 23 July 1972, resulting in a flood of data. Acutely aware of this need in order to obtain and preserve operational funding, NASA officials undertook extensive efforts to encourage both domestic and foreign users of this data (Mack, 1990; Maher, 2019).^19^ These efforts included the publication of *Mission to Earth: Landsat views the world* (1976), in which data collected from Landsat’s multispectral scanner was used to construct a series of visually arresting colour plates depicting biological and geophysical features across the planet. These were used to promote practical applications in: agriculture, forestry and range resources, land use and mapping, geology, water resources, oceanography and marine resources (Short et al., 1976, pp. 2–4).^20^ Although *Mission to Earth* offered numerous technical explanations of the satellite and its instruments and how its data could be used for practical purposes, the book was also intended for general audiences. The images themselves comprised its central and most fetching feature. The 400 plates of Landsat images contained, according to the authors, ‘a richness in color and form that rivals the finest of the French impressionist paintings’ (Short et al., 1976, p. v).

Many of Wilson’s ideas for fortifying an oceanographic remote sensing program at NASA closely aligned with the data aesthetics of *Mission to Earth*. More generally, Wilson wanted scientists to get away from information clutter, to escape the habit of representing data in ways that were neither self-evident nor self-explanatory, in ways that required too much additional text to make clear.^21^ The need to declutter extended not just to the presentation of scientific data, but also to the promotion or marketing of scientific research programs, including the nascent ocean remote sensing program at NASA. An example of the kind of promotional material Wilson wanted to get away from came from the International Geophysical Year (IGY). The IGY was a worldwide interdisciplinary scientific endeavour, that ran from July 1957 to December 1958, to collect coordinated geophysical data on the Earth’s oceans, lands and atmosphere. Paul Edwards identifies the IGY as a pivotal moment in the history of global knowledge construction due to its overarching aim to treat the planet as a ‘single physical system’ amenable to scientific study (Edwards, 2017, pp. 36–37; GARP JOC, 1969, p. 15). However, Aronova (2017) argues that the IGY was more visionary than practical, at least in its immediate effects, since its comprehensive data collection efforts were stymied by their storage in analog format on microfilm, a format unusable in numerical models running on digital computers. Even if impractical, the IGY was global in conceptual scope and an important precursor to later attempts to study and understand the planet as an interconnected system.

**Figure 1.**
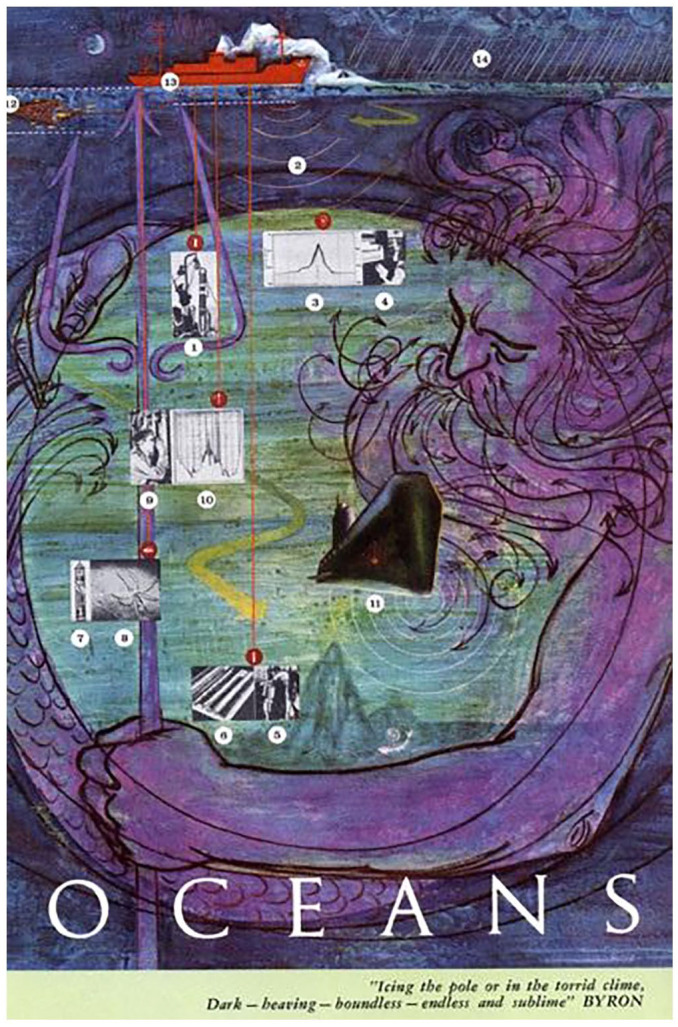
NAS International Geophysical Year poster (1958) depicting scientific activities in the world’s oceans (reprinted with permission from the National Academy of Sciences, courtesy of the National Academies Press, Washington, DC).

The posters promoting the IGY were, in Wilson’s view, beset by information clutter from the outset. Understanding these posters required an exercise in semiotic decoding reminiscent of Roland Barthes’ *Mythologies* (1972). Mythical figures like Poseidon and inspirational quotations from Romantic poets, the Bible and Leonardo da Vinci all framed the space of the posters. On each poster, different regions of the Earth were depicted along with a number of smaller images representing the different kinds of scientific research being undertaken. Exactly what was being represented was not always clear, so each small image was numbered, and each number referred to a passage in an accompanying interpretive booklet (see, for example, Figure 1).^22^ Whatever their artistic merits, in Wilson’s opinion, the IGY posters were cluttered assemblages of information that were neither intuitive nor straightforward to the viewer.

Against this background of problems with data and project communication, in the early NASA and the Jet Propulsion Laboratory (JPL) contracted Stevens’s newly formed INI 1980s. The immediate task was to build a community of satellite data users by making the data from the Seasat and Nimbus-7 satellites intelligible to scientists not yet using that data in their research. Wilson wanted to develop images containing satellite data that could be understood at a glance, with little need for further elaboration and certainly no need for an explanatory booklet. In alignment with the aesthetic style of *Mission to Earth*, InterNewtwork transformed data collected from instruments on space-based platforms into simple colour-coded schemes superimposed onto global or regional images of the planet. Perhaps influenced by the visual presentation of data in *Mission to Earth*, INI used techniques akin to the principles of ‘graphical excellence’ proposed by statistician and political economist Tufte (1983) in *The Visual Display of Quantitative Information* to turn satellite data into visually compelling material for both scientists and the general public.

**Figure 2.**
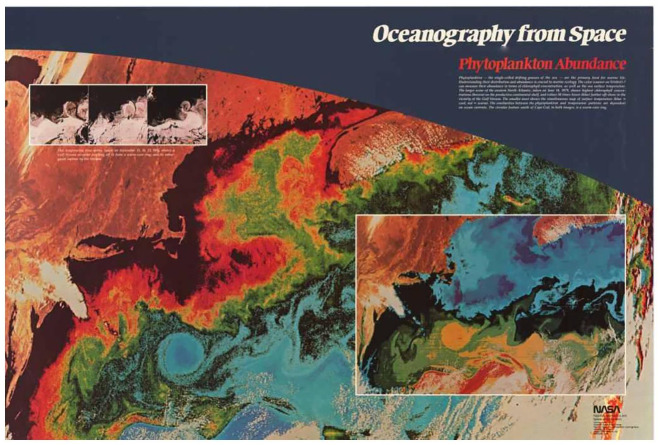
NASA’s Oceanography from Space poster for ‘Phytoplankton abundance’ (1983) as designed by Payson Stevens’s InterNetwork graphic design company (reprinted with permission from NASA; design by InterNetwork, Inc.).

The final products of Stevens’s NASA/JPL oceanography project were five colour posters with blue/slate backgrounds that depicted oceanographic satellite data for: Antarctic sea ice, marine winds, phytoplankton abundance, sea surface structure and sea surface topography. With their ocean-like blue/slate colours, each poster was readily identifiable as part of the series NASA called ‘Oceanography from Space’. The poster of ‘phytoplankton abundance’, for instance, depicted satellite data from Nimbus-7’s Coastal Zone Color Scanner, a multi-channel radiometer that measured solar radiation reflected from the Earth’s surface. The levels of ‘green’ detected by these measurements indicated the concentrations of chlorophyll on or close to the ocean’s surface. On the poster, chlorophyll levels were colour-coded and mapped onto an image of the mid-Atlantic Ocean off the eastern coast of the U.S. (Figure 2). Representing data in this way revealed not only the higher concentrations of phytoplankton on the continental shelf, but also the broad contours of the Gulf Stream as phytoplankton were swept along by ocean currents. These patterns ‘popped’ out of this visual presentation in a way that traditional tables of data could not capture.

In an article in San Diego’s *Tribune* from 1983, Stevens described his approach to merging science and art in the posters: ‘A new gallery of artistic ocean imagery is evolving, and scientists are generating its works. They use satellites as their brushes, computers for canvas and statistics for paints. The satellites gather the images, the computers add colors.’^23^ By combining scientific data with an artist’s aesthetic sensibility, Stevens could present complex data sets in a way that made them immediately intelligible and attention-grabbing to expert and general audiences alike. Stevens’ work on the Oceanography from Space posters provided him with experience in the attractive display of scientific information. The work would get him noticed by the ESSC and it would contribute significantly to his creation of a visual ‘brand’ for Earth system science.

## The ESS brand experience

The ESSC hired Payson Stevens and INI in 1985. As seen above, at least some committee members were conscious that the ESSC had a messaging problem. The early draft materials fell far short of the ‘sales goal’ of providing a consistent messaging for ESS that would be compelling to civil servants, politicians and the public, while also maintaining fidelity to the underlying science. Stevens, fresh from his success promoting NASA/JPL’s ocean remote sensing program, was an obvious fit with the ESSC’s project, which would also rely heavily on satellite data. In early January 1986, Stevens outlined his idea to aesthetically link all ESS material in a proposed ‘promotional strategy’. According to Stevens, all brochures, reports, posters, folders, postcards and convention displays ‘should be married to each other through key design elements (color for program; logo; typeface; etc.) much the way the “Oceanography From Space” products were’. Visually linking together key design elements was a crucial branding technique that emerged in the 1980s (Moor, 2007). Stevens even recommended a series of posters ‘analogous to the “Oceanography From Space” series’.^24^ The glossy images included as part of the *Closer view* report folder released in January 1988 depict satellite data (e.g. sea surface topography, Antarctic ice, air-sea interactions, stratospheric ozone) superimposed on maps of part or all of the Earth’s surface, similar to the images produced for the Oceanography From Space contract (NAC, 1988).

In developing a promotional strategy for ESS, Stevens was clearly drawing heavily on techniques that made the Oceanography from Space project successful. But Stevens was not just suggesting a similar approach to envisioning information. Rather, he saw the new ESS project as being concretely linked with the Oceanography from Space material. Stevens had two different NASA contracts, but he did not see those contracts as being separate, self-contained projects. He saw them together as part of a single, larger project. He deliberately developed the ESS theme as a continuation of the Oceanography from Space colour scheme to reflect what he saw as a continuity between the two projects.^25^ Others involved with the ESSC’s work similarly understood and voiced the importance of aesthetic continuity between the Oceanography from Space posters and ESS material. A NASA official defended an extension of Stevens’s ESSC contract specifically because of the potential for cohesion between ESSC outputs and previous work done by INI: ‘It is desirable to maintain continuity in these publications to aid public association of these efforts – clearly the unique and successful style of design used by INI is not capable of duplication elsewhere.’^26^

**Figure 3.**
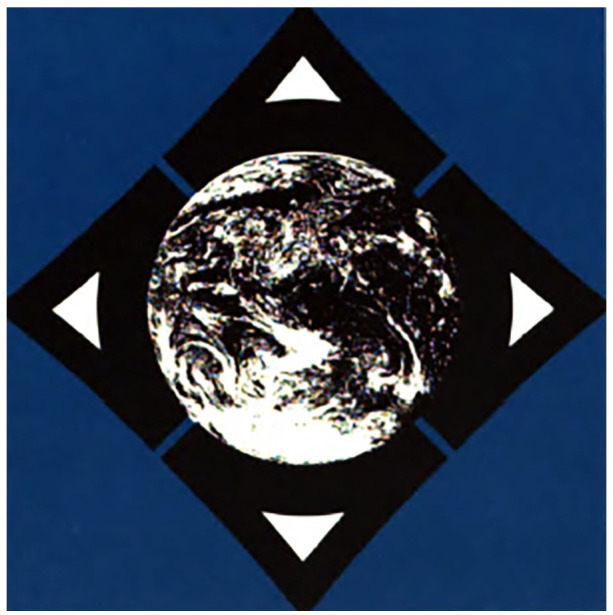
The ESS logo is on all ESSC reports and other media products (reprinted with permission from NASA; design by InterNetwork, Inc.).

In his work for the ESSC, Stevens developed a distinctive ESS patterning of visual information. This would make everything produced by the ESSC immediately recognizable and embedded with the values of environmental concern, interdisciplinary research and an interconnected planet. Thematic unity was created by using a common colour scheme, standard layout and repeating images. The central figure of this new scientific brand was the development of an ESS ‘logo’, often a key part of branding strategies (Figure 3). In the middle of the ESS logo is a picture of the Earth. The recognizable cloud patterns suggest that it is a gently edited version of the famous photograph taken on 7 December 1972 by the crew of Apollo 17 as they sped towards the moon on the last Apollo flight. The picture, commonly called ‘Blue Marble’, is noteworthy because it was one of the first – and certainly the most well-known and reproduced – colour images of the planet (Poole, 2010). In the ESS logo, the dark void of space around the Earth is reshaped into four separate arrow points, each with a triangular white middle section. The thick black lines of the arrows may allude to the lines of a system flowchart (see below). The four arrows suggest the totality of the Earth system by gesturing at either the four cardinal directions or the four major parts of the Earth system (i.e. the atmosphere, biosphere, hydrosphere and lithosphere). The logo appeared on all of the ESSC’s published materials and even on ESS t-shirts that Stevens had printed and planned to sell at ESS conference displays.^27^

**Figure 4.**
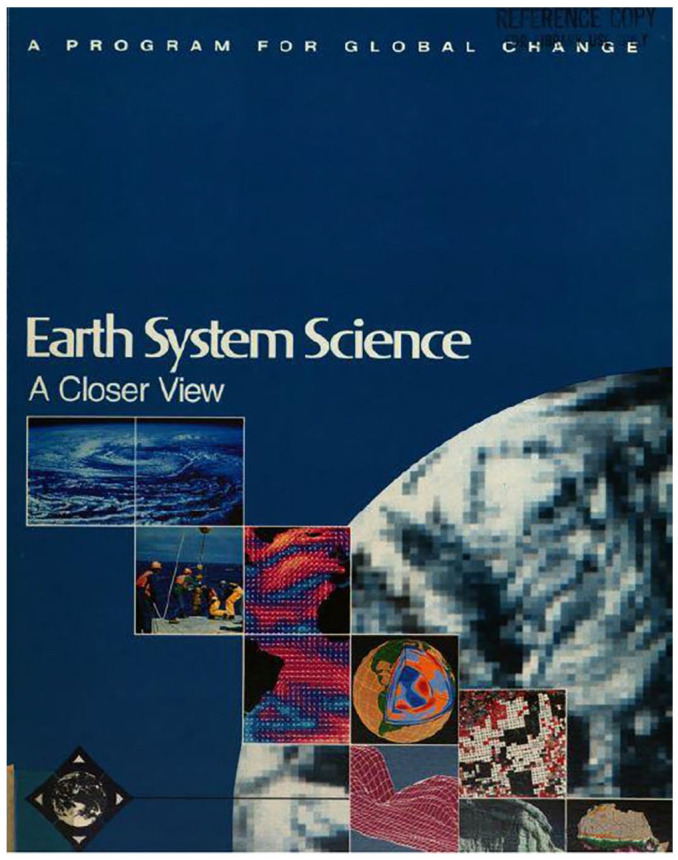
The ‘ESS Blue’ serves as the background colour for all ESSC material, including its *Closer view* (1988) report (reprinted with permission from NASA; design by InterNetwork, Inc.).

The most striking feature of ESS branding was its distinctive blue colour. Why blue? The reason is not explicit, but it might be because the Earth appears mainly blue when viewed from space in the visible light spectrum.^28^ The specific blue selected by Stevens for ESSC material was Pantone Matching System (PMS) 301 Blue.^29^ PMS is a proprietary system used by professional designers and others to maintain colour fidelity across a variety of materials, physical and digital. PMS-301 Blue permeated all ESSC materials. It was the background colour for both the *Overview* and *Closer view* reports (Figure 4). It was on ESS posters and presentation slides. It was the colour of the folders in which materials were packaged for the ESSC press conference and mass mailings. The ESSC’s letterhead used this blue, and it was even the background colour for t-shirts bearing the ESS logo. Members of the ESSC knew that the blue was a distinct and important feature, variously describing it as their ‘trademark blue’, ‘our blue’ or simply ‘ESS blue’.^30^

It was not enough to simply create an ESS brand. By itself, a brand does very little. There also needs to be a strategy to promote the brand so that it is recognized by relevant constituencies. Stevens’ promotional plan centered around the release of the *Overview* report in spring 1986. Writing to Shelby Tilford, then Director of Earth Sciences at NASA’s Office of Space Science and Applications, Stevens emphasized that:

It is important to have an overall strategy for the release of information to further ESS. What is needed (as they say in the business) is an orchestrated campaign which is coordinated with key political events and reaches specific targeted audiences. … [It is] important that we move immediately to identify key dates throughout 1986 (and beyond) and coordinate specific media releases to promote ESS.^31^

He also provided a ‘laundry list of potential products which can be considered important elements for reaching a wide range of audiences’ that would supplement the ESSC’s scientific and technical reports. The list included a number of promotional products that would share ESS’s core thematic and chromatic elements. Postcards would highlight research and hardware that were ‘beautiful enough to save and pin up’. ESS posters, similar to those done for the Oceanography from Space project, would ‘draw interest/recognition to [the ESS] program at scientific meetings like those of the American Geophysical Union (AGU) and the American Association for the Advancement of Science (AAAS). Pieces in glossy science magazines, like *OMNI* and *National Geographic*, would introduce the ESS project to public consumers of science. More public attention might come from sending promotional releases to journalists ‘as potential storylines to whet their appetites’. Promotion would not be restricted to print media. A 10-to-15-minute video piece using ‘state-of-the-art computer graphics’ and an audio-visual slideshow was to be created.^32^ Of course, not all these ideas to promote ESS came to fruition, but Stevens had a broad vision for using a variety of media to create excitement and buzz.

The ESS promotional strategy reached its pinnacle on 26 June 1986 at NASA Headquarters, when the *Overview* report was publicly released at a widely advertised press conference. The 50-page report outlined the Earth system science research program recommended by the ESSC. (More detail would be added two years later when the *Closer view* (1988) report was released with much less fanfare.) This was the press conference to launch the ESS project and give it broad public attention. ESSC members wanted to make a ‘big splash’ among scientists, politicians and the public.^33^ Planning for the public launch began early in 1986. The press conference would attempt to sell ESS by offering what was arguably a science-brand experience, akin to the brand experiences created in stores by companies like Disney and Nike (Klein, 1999, pp. 56, 149–152; Moor, 2007, p. 48). All of Stevens’ communications savvy was put to use in the effort to create a lasting impression and generate interest. The six months of preparations leading up to the event were hectic. The report and other press materials, all branded with the ESS logo and colour, had to be made ready for printing and distribution. The layout of the presentation room had to be designed to properly showcase ESS. Arrangements had to be made for the heads of NASA, the National Oceanic and Atmospheric Administration (NOAA) and NSF to speak at the conference, and the conference had to be so timed to allow members of Congress to attend. This amount of work was beyond any single individual, so the ESSC enlisted the help of NASA Headquarters’ Public Affairs Officer Jim Kukowski. Kukowski expertly generated media attention by coordinating multiple press releases and directly contacting news outlets like *Time Magazine*, NBC and *USA Today*. He also did the mundane work of arranging name tags and having an ESS lapel button made for ESSC chair Bretherton.^34^

On the day of the conference, CNN was there to provide live coverage. It was intended to be a ‘classy’ affair. ESSC manager McCauley reportedly wore a ‘teal’ suit to the event, keeping with the project’s colour scheme.^35^ On the wall was a large, Stevens-designed ESS poster with the distinctive blue background dominated by a large version of the ESS logo. Superimposed onto the Earth at the center of the logo was what the ESSC called an ‘Earth system wiring diagram’ (see below). Surrounding the logo and diagram were images of a satellite, a forest, a volcano erupting and satellite data for wind directions mapped onto an image of the Pacific Ocean. At the bottom was the well-worn quotation from T.S. Eliot: ‘We shall not cease from exploration/And the end of all our exploring/Will be to arrive where we started/And to know the place for the first time.’^36^ Images from the *Overview* report were mounted on foam boards along the walls of the conference room.^37^ Press kits were fully branded and contained copies of the *Overview*, an ESS fact sheet, slide images and a copy of Bretherton’s presentation. Documents were placed on display at the back of the room with ‘stacks below for the taking’. The logos for ESS, NASA, NOAA and NSF were ‘everywhere’.^38^ A select group of scientists, politicians and media representatives heard remarks from the heads of NASA, NOAA and the NSF as well as from Bretherton, who spoke for 20 minutes with a thick stack of slides carefully prepared by Stevens and emblazoned with the ESSC’s trademark blue and logo.^39^ Whether individuals in the room looked closely at the images on the walls, listened to the presentations or read any of the printed material, they were inundated by the ESS brand.

The recurrence of exactly the same tone of blue across all media, combined with the logo and layout elements, all helped project ESS beyond the conference room. The coherent design elements assembled by Stevens gave viewers a means of identifying diverse media as all part of the ESS project. Since all reports, letters and presentations were marked with the ESS logo and coloured with PMS 301 blue, they could all be easily identified as ESS products. To recognize the coherent aesthetic style was to immediately recognize ESS. In the same way that corporate brands are metonyms that manage diverse elements, the aesthetics of ESS linked together satellite remote sensing, computer modeling, interdisciplinary Earth science research, environmental concerns and an interconnected planet, the ‘Earth system’.

## Unintended consequences: Conceptualizing the Earth system

All of the ESS material, in one way or another, gestured holistically at an interconnected planet: The recurring picture of the whole Earth, the cardinal coordinates of the logo and the trademark blue linking ESS to a view of the Earth from space. In the poster Stevens designed as the centerpiece for the June 1986 press conference, a flowchart of the Earth system was superimposed on a version of ‘Blue Marble’ and framed by the cardinal points logo. This specific juxtaposition of overlaid images arguably suggests that the real Earth and the diagram are in close correspondence and perhaps interchangeable. The diagram could be interpreted as either a literal or metaphorical blueprint of the actual processes taking place on the Earth. Though never explicitly recognized as part of the ESSC’s promotional strategy, what the ESSC called its ‘wiring diagram’ (and what was later called the ‘Bretherton diagram’, Figure 6) forcefully conveyed ESS values and captured what was meant by the ‘Earth system’.

The diagram was a flowchart of the movement of material and energy through the Earth’s atmosphere, waters, landmasses and biota. On the left side of the diagram, the sun and volcanoes were external components that acted physically and chemically on different parts of the Earth’s atmosphere. The atmosphere, in turn, effected changes in the chemistry and physical dynamics of the oceans as well as terrestrial ecosystems. On the right side of the diagram, ‘human activities’, like the release of carbon dioxide and ‘pollutants’, would feed back through the components of the Earth system as climate change.^40^ In a glance, the diagram conveyed a crucial aspect of the ESS brand, the idea there was an ‘Earth system’ composed of many interconnected subsystems awaiting interdisciplinary scientific study. The inclusion of human activities added urgency by highlighting the potential for detrimental human-driven changes to the system.

The Bretherton diagram was a simplified version of a more complex diagram the ESSC developed at the start of its work. With roots in depictions of nutrient cycles and interconnected climate processes from the 1960s and 1970s, both ‘wiring diagrams’ also resembled flowcharts because a central long-term aim of ESS was to develop a computer model of past and present planetary conditions that could (hopefully) predict future states.^41^ The model was to be like contemporary weather and global circulation models, but more far-ranging (Edwards, 2010). Thus, the original ‘wiring diagram’ was conceived as a schematic of the Earth system serving as a conceptual steppingstone to an eventual computer model. Developing even a provisional schematic of the Earth system for this purpose required addressing a number of conceptual problems confronting the ESS program. Central among these was the question of timescale. The whole Earth was the spatial scale of the project, but the appropriate timescale was less clear to the members of the ESSC’s Earth System Modeling Working Group.^42^ The issue was that the timescales for Earth processes range from the comparatively short (lasting seconds, days, weeks or years, as for atmospheric turbulence, seasonal vegetation cycles and global weather systems), the more intermediate (lasting decades to centuries, as for soil erosion, climatic conditions, carbon dioxide variations, ocean circulation and nutrient cycling), to the extremely long (occurring over thousands to millions of years, as for glacial periods, soil development, speciation and plate tectonics). These widely varying processes could not be practically brought together in a single, unified model (NAC, 1988, p. 27).

**Figure 5.**
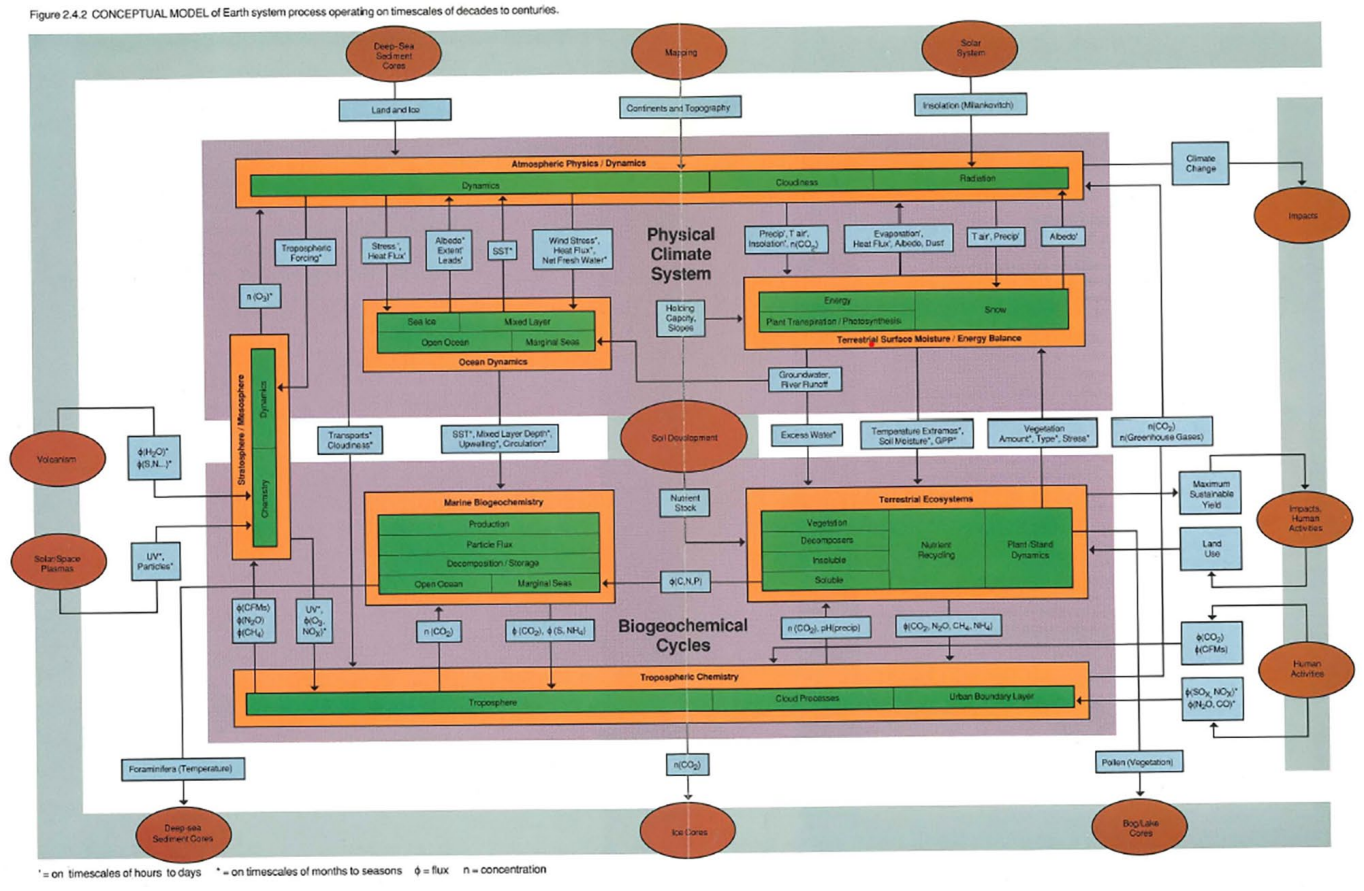
The more complex Earth system wiring diagram from the ESSC’s Closer View (1988), depicting Earth processes occurring on timescales of decades to centuries (reprinted with permission from NASA; design by InterNetwork, Inc.).

After much discussion and debate, the Working Group concluded that the aim of building a comprehensive Earth system computer model put practical constraints on how the Earth system would be conceptualized. For the sake of current and near-future computational capacities, the group decided that ESS should focus on the timescale of decades to centuries, the timescale most relevant for human lifespans (NAC, 1988, p. 15). Even with the project’s scope significantly circumscribed, the resulting diagram of the Earth system concept was complicated (Figure 5). Large purple rectangles represented the two predominant subsystems at the decades-to-centuries timescale: the physical climate system and the biogeochemical cycles system. Orange and green boxes indicated different subsystem processes. These were linked together by arrows that pointed to the flows of matter and energy between the different subsystems. Brown ovals stood for processes external to the Earth system, but by which materials and energy could enter or leave the system.

**Figure 6.**
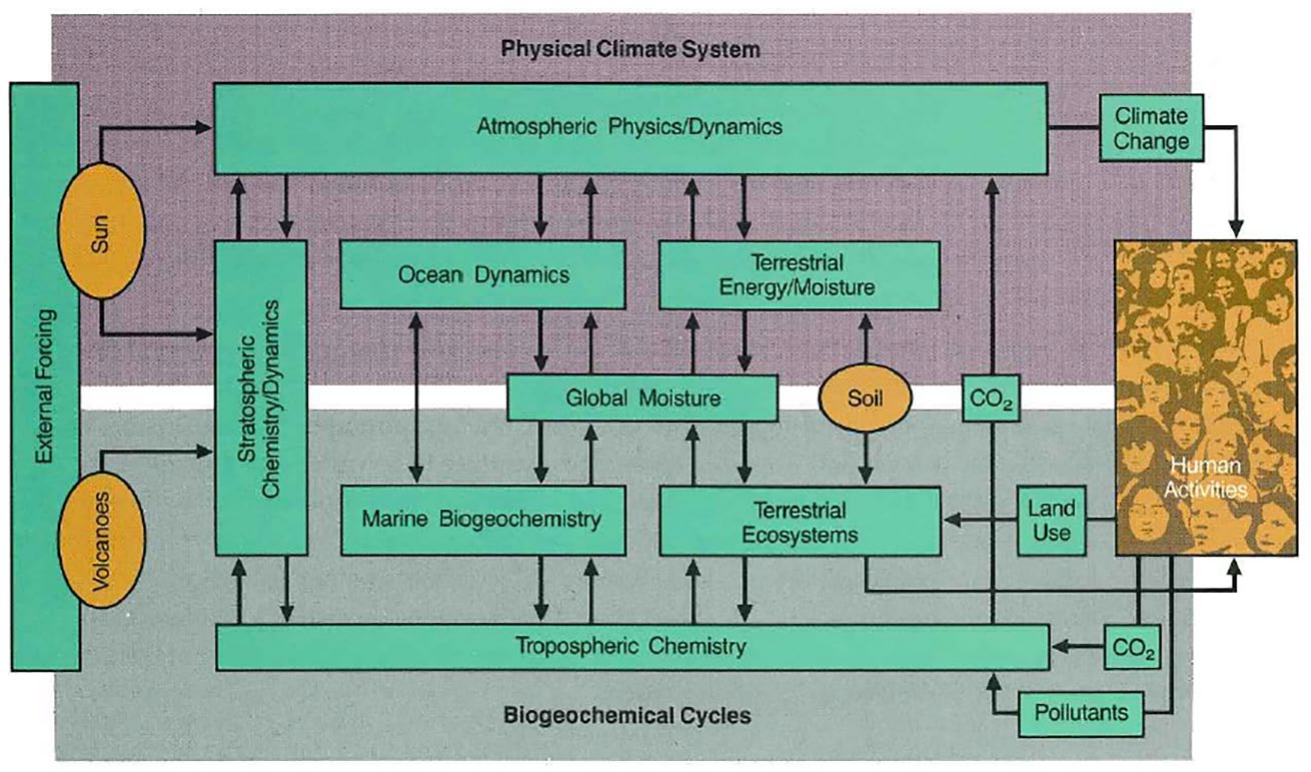
The simplified Earth system wiring diagram, for timescales of decades to centuries, from the ESSC’s Overview (NAC, 1986) report, now referred to as the ‘Bretherton diagram’ (reprinted with permission from NASA; design by InterNetwork, Inc.).

This wiring diagram may have done the conceptual work of capturing the key components of an Earth system model on the timescale of decades to centuries, but it was complex and had a level of detail that made it not immediately understandable to many viewers. Recognizing that this might conflict with the need to promote the program, the ESSC developed a simplified version of the wiring diagram (Figure 6).^43^ It was this simplified diagram that would be superimposed on ‘Blue Marble’ on the ESS press conference poster designed by Stevens. The diagram would become emblematic of ESS (Dry, 2019, p. 266; Lenton, 2016, p. 14). What made this diagram compelling was clearly not its fine-grained fidelity to the ‘science’ goals of the ESSC. Like Galileo’s depictions of the moon’s surface in *Sidereus nuncius* (1610), the Bretherton diagram did not aspire to a realistic or isomorphic mapping between the wiring diagram and the world (Edgerton, 1984, pp. 228–229; Vertesi, 2014, pp. 17–21). Gone were the multicoloured subsystems boxes and details about the fluxes of matter and energy that linked the subsystems together.

What was left were fourteen plain blue/green boxes, each containing a simple one-to-three-word description of a planetary subsystem with thin, black arrows pointing in the direction of system flow. The simplified diagram did what the complex diagram could not: It offered an easily comprehended, visual encapsulation of the Earth as a system of interconnected subsystems. The diagram further showed how the Earth system could be scientifically studied with interdisciplinary collaboration. Each box represented a planetary subsystem, but also a different Earth science discipline, with the arrows pointing to connections between subsystems and, by implication, the interconnectivity among Earth science disciplines. Earth scientists looking at the simplified diagram could immediately see roughly where their disciplines contributed to the overall project of Earth system science.

For the ESSC, the main point of the simplified diagram was that it ‘provides both a motivation and a mechanism for promoting interactions among specialists in different disciplines’ (NAC, 1988, p. 31). Thus, the Bretherton diagram was an essential part of the ESS brand, even more than the ESS logo and PMS 301 blue. It served as a heuristic tool for generating consensus about studying the Earth as a system and provided a conceptual framework for interdisciplinary cooperation among Earth scientists. Bretherton described how the diagram could be used to demonstrate how the different disciplines fit together in the larger ESS framework:

[The wiring diagram] became useful because, from there on, every time I was talking about this program to any group of scientists … [t]he message was, ‘this is where you fit in! Your community, your effort – you can only help with this aspect of the measurements or this aspect of your modeling’, which enabled us to infer some things which we can’t measure, but nevertheless information that’s needed to say how the system as a whole will look, rather than just the fragmented pieces of it. (Goldstein, 2007, p. 134)

This abstract diagram acted as a shorthand for how disciplinary research on the Earth system could fit into a comprehensive and integrated framework. Bretherton could, say, point to the subsystem box labeled ‘ocean dynamics’ to show a physical oceanographer where their work fit into the larger interconnected system and research project. Likewise, he could point an ecologist to the ‘terrestrial ecosystems’ box or an atmospheric chemist to the ‘tropospheric chemistry’ box. The diagram was a social and scientific integrator, taking in disparate disciplinary pieces and returning a large-scale, cohesive, interdisciplinary ESS research program. The diagram’s abstraction, rather than its scientific realism, made it an effective tool to convey the meaning of the ‘Earth system’ to diverse audiences as well as a device to build interdisciplinary solidarity in Earth science communities. Scientific representations like the Bretherton diagram are ‘social achievements’ insofar as they are the end-product of group organization and agreement (Hepler-Smith, 2017, p. 123). But they can also do ‘social work’ by creating new communities or enlisting existing ones (Vertesi, 2014, p. 244). Scientists use visual representations – be it Galileo’s lunar landscape, Mars Rover images or the Bretherton diagram – to build communities of like-minded individuals that coalesce around a particular set of beliefs, goals and practices, often in advance of any actual scientific research being completed (Vertesi, 2014; see also Latour, 1990).

Despite all the promotional efforts, the specific ESS project never gained widespread traction. It was never implemented on the grand scale originally envisioned, though components were taken up by NASA’s Mission to Planet Earth (MTPE) that received initial Congressional approval and appropriations in 1989.^44^ The reasons ESS failed to launch in the mid-1980s flowed mainly from a lack of consensus among Earth Science communities. For many in the geological community, ESS focused too narrowly on Earth processes occurring on the shorter timescales of decades to centuries. This was at the expense of much longer-term Earth processes that geologists and geophysicists typically studied. Other Earth science constituencies – including some ecologists along with geologists and geophysicists again – were reticent because the project’s emphasis on observations from space might limit funding to research areas less reliant on or able to use satellite data (Barton, 2020). Some scientists (and politicians) expressed the reservation that ESS’s massive price tag threatened to absorb all available government research dollars. Still, others were concerned that implementing a network of large satellite platforms would take too much time when the need for research data was immediate (Conway, 2008, pp. 247–248). Given the project’s enormous ambition in a period of declining government research funding, it is perhaps not surprising that the ESSC failed to generate a robust consensus even among Earth scientists themselves.

Though ESS as a specific research program failed to gain traction in the 1980s, the Earth system, as depicted in the Bretherton diagram, became one of the ESSC’s most enduring legacies. Among other places, the Earth system concept was adopted by the International Geosphere Biosphere Programme (IGBP), a research program approved by the International Council of Scientific Unions in 1986 to quantitatively investigate interactions between the Earth’s biosphere and its physical climate processes (Conway, 2008, p. 233). Kwa (2005) notes that the ESSC preceded the IGBP, though there was membership overlap between the two groups. By 2000, former ESSC (and its Earth System Modeling Working Group) member Berrien Moore was chair of the IGBP. In the IGBP’s *Global Change Newsletter* for that year, the ‘Earth system’ is mentioned 77 times in the issue’s 20 pages. Notably, this issue also contains an article by Paul Crutzen and Eugene Stoermer suggesting that humanity had entered a new geological era – the Anthropocene – due to the many profound changes that humans had wrought on the planet through the burning of fossil fuels and the release of carbon dioxide into the atmosphere (Crutzen & Stoermer, 2000). While historians have yet to comprehensively study the importance of the IGBP, the idea of the Anthropocene that first emerged from the pages of its newsletter has experienced huge uptake in both scientific communities and the humanities.^45^

The far-ranging traction of the Earth system is arguably largely attributable to the Bretherton diagram. Kwa argues that the ESSC’s work became ‘especially famous’ because of the Bretherton diagram: ‘It was useful because the diagram implicitly identified the sciences that were to be included in a global research programme and assigned a meaningful role to each of them’ (Kwa, 2005, p. 928). By 2009, when NASA’s History Office conducted interviews with some scientists and NASA officials associated with ESS, many had forgotten or misremembered key events and details. Despite this, the one thing that almost all actors reported was the significance they placed in the Bretherton diagram. One individual – solar-terrestrial physicist and ESSC member Lennard Fisk – went so far as to assert, somewhat bizarrely, that the ESSC had produced no reports, at least none he had ever been able to locate, ‘[b]ut the Bretherton Wiring Diagram has lived forever’. Fisk described it as, ‘a one-stop shopping diagram that showed how the Earth worked and how NASA’s missions to address this were going to satisfy the science that needed to be done by the Bretherton Wiring Diagram. It was a very clever diagram as a result’ (Fisk, 2010). More recently, Timothy Lenton – a self-described Earth system scientist, Director of the Global Systems Institute at the University of Exeter and author of *Earth system science: A very short introduction* – proclaims the Bretherton diagram to be the ‘most lasting legacy’ of the ESSC. According to Lenton, the Bretherton diagram provides the ‘social glue’ for interdisciplinary studies of the Earth by putting ‘a whole range of existing scientific subjects – and their associated scientific communities – together on the same map’ (Lenton, 2016, p. 14).

## Conclusion

The ESS project was ambitious, arguably too ambitious given the political economy of U.S. science by the mid-1980s. U.S. government funding for large-scale research was diminishing, compared with its peak in the 1960s. The ESSC was not blind to this context. The committee clearly understood that economic resources were scarce and that its project faced a struggle for existence. This is evident in the way that members ongoingly emphasized the importance of ‘selling’ ESS. The same members were also much aware that, outside their immediate community, their aspirations risked sounding inflated – like proclamations of ‘Guinea Pig Week’. To better communicate its proposed research program, many of the techniques the ESSC adopted cohered with those of a corporate management tool that had itself taken on a new character: branding. The ESSC tried to manage scientific practices and funding by using rhetorical and aesthetic tropes that closely resembled those of corporate branding to promote its values and goals.

Payson Stevens’s work helped transform the ESS products and various communications strategies into a science brand. The ESS brand – with its distinctive blue colour and abstract logo organized around a version of the iconic ‘Blue Marble’ photograph – communicated the ideals of a research program oriented towards the study of the whole Earth as a unified and integrated system. This reinforced the ESSC’s efforts to conceptualize the ‘Earth system’ using a wiring diagram. The complex wiring diagram detailed the key relationships between the sun, atmosphere, biosphere, hydrosphere and lithosphere that would eventually be incorporated into a computer model of the Earth system. However, as Bretherton himself acknowledged, the purpose of the simplified wiring diagram was not modeling. Its purpose was managerial and organizational. The simplified wiring diagram, what came to be called the Bretherton diagram, was a tool to help Bretherton and other ESSC members sell ESS. It helped Earth scientists ‘buy into’ the project by showing where their disciplines’ research fit within the ESS program. The Bretherton diagram was never intended to be a realistic scientific model of the Earth system, but was a management tool, an organization chart for coordinating the interdisciplinary study of the Earth system across Earth science communities. The overlay of the Bretherton diagram with the ‘Blue Marble’ photograph on some ESS posters alluded to the interdisciplinary study of the Earth as well the Earth system concept more generally.

The ESSC’s branding efforts might be considered a failure since the specific program it promoted never reached fruition. However, branding did achieve one significant and lasting effect: It entrenched the nascent concept of the ‘Earth system’. Use of the phrase exploded in 1986, the year that the ESSC held its press conference and released its first major report. This is evident in Google’s n-gram viewer, which clearly shows the use of the phrase ‘Earth system’ taking off dramatically in the mid-1980s.^46^ Likewise, *Science* and *Nature*, two of the most prominent non-specialist scientific journals, both show overwhelming increases in use of the phrase after 1985. *Science* has 820 hits post-1985 and only 23 hits for the entire preceding period, while *Nature* has 2,407 hits after 1985 and only 43 hits before.^47^ After 1986, the ‘Earth system’ became ubiquitous in the Earth sciences as *the* common phrase used to express the concept that the Earth is a number of interconnected systems. The concept is now so familiar and commonplace that it feels ‘natural’. When it is invoked, it requires little explanation or justification. For most Earth scientists, it is now undeniable that the Earth system was always already ‘out there’ in the world awaiting discovery. Yet, the concept’s roots are clearly traceable to the promotional work done by the ESSC.

While the specific ESS research program proposed by the ESSC did not gain widespread support in the 1980s, Earth system science has gained some traction more recently. In 2016, editors at Oxford University Press considered ESS important enough to warrant a slender volume in its *Very short introductions* series (Lenton, 2016). NASA continues to describe its research on climate change using satellite observation platforms as ESS.^48^ A small number of universities offer graduate training in ESS. Only a few academic journals have ‘Earth system science’ in their titles, but the phrase is now commonplace within research articles as well as in books and textbooks on climate change and the Earth sciences.^49^ Today, scientists who self-identify as doing Earth system science tend to define their discipline as grappling with anthropogenic environmental issues on the global scale, especially climate change. Scientists like Will Steffen, Timothy Lenton, and Johan Rockström frequently link their Earth system research to the ‘Anthropocene’, a term used in the sciences and humanities to define the historical period characterized by the global effects of human industry. They focus on the interconnecting feedback loops with potential ‘tipping points’ that might lead to new planetary conditions less hospitable to humans (Lenton, 2016; Rockström et al., 2009; Steffen et al., 2020).

Recent trends in ESS have only a passing resemblance to the specific project conceived by the ESSC in the mid-1980s. It is noteworthy that the elements of the ESSC’s grand design that have endured are precisely the elements that were captured by what this paper calls the ESS brand. This branding evoked and entrenched the key values of Earth system science: a unified planetary system, interdisciplinary science and environmental change. This may not have been the success hoped for by the ESSC or NASA, but the persistence of ESS and the ‘Earth system’ are testaments to the compelling potency of what may now be described as the ESSC’s branding campaign.

## Notes

1. YouTube. Apple 1984 Super Bowl Commercial Introducing Macintosh Computer. Available at: https://www.youtube.com/watch?v=2zfqw8nhUwA (accessed 31 January 2021).

2. Dutreuil – in line with James Lovelock himself – argues for a strong, direct connection between the Gaia hypothesis and Earth system science (Dutreuil, 2020; Lovelock, 2009). According to Dutreuil, some contemporary researchers (notably Timothy Lenton) identify as Earth system scientists and trace their scientific and intellectual lineages to the Gaia hypothesis. While some may make this connection, my research suggests that the link between the Gaia hypothesis and Earth system science is more philosophical than historical. Further, whether this connection is plausible or not will likely depend on the interpretation of the Gaia hypothesis adopted. For instance, interpretations that argue that biota modify the abiotic environment to produce optimal conditions for life were and remain controversial in scientific communities. Other interpretations that are less teleological are less controversial (Kirchner, 1991; Ruse, 2013). As this article and other research shows, the historical roots of Earth system science are traceable not to Lovelock’s Gaia hypothesis but rather to the institutional work done by NASA’s Earth System Sciences Committee (ESSC) in the early-and mid-1980s that was attempting to capitalize on broader post-WWII trends in Earth science communities regarding the need to study the planet in more interconnected ways (Barton, 2020; Conway, 2008). Lovelock’s ideas may have been known to the members of the ESSC, but there is no archival evidence to suggest that the Gaia hypothesis played a significant role in the ESSC’s attempt to develop Earth system science (ESS), a large-scale research program to study the planet as an interconnected system with a heavy reliance on satellites and computer modeling.

3. John Wainwright notes that there was no hydrologist on the committee as none that were identified to serve on the committee were available (Wainwright, 2009, p. 149).

4. This included but was not restricted to: the National Oceanic and Atmospheric Administration (NOAA), the National Science Foundation (NSF), the United States Geological Survey (USGS), the Department of Agriculture, the Department of Energy, the Office of Naval Research, the Environmental Protection Agency, the Agency for International Development, Housing and Urban Development, the Department of State and the Department of Defense.

5. ESSC Working Framework, Sep 1984, ESSC/Bretherton Committee File, Box 18042, NASA HRC, Washington, DC.

6. Notes, n.d., ESSC/Bretherton Committee File, Box 18042, NASA HRC, Washington, DC. Mamet’s *Glengarry Glen Ross* made its world premiere at the National Theatre in London in September 1983, and then premiered on Broadway in March 1984. See: Josh Ferri (2012) Expletives, Awards and Star Power: Why *Glengarry Glen Ross* Sells as a Modern American Classic. Available at: https://www.broadway.com/buzz/164979/expletives-awards-and-star-power-why-glengarry-glen-ross-sells-as-a-modern-american-classic/ (accessed 19 April 2022).

7. Indeed, it was in part this past ‘blandness’ that created the need for the ESSC’s work in the first place. There had been previously published ‘bland’ reports from the NAS on incorporating space technologies into Earth science research (Committee on Earth Sciences, 1982, 1985) and formed the starting point for the ESSC’s work (NAC, 1988, p. 195).

8. Letter from [John Dutton] to Francis [Bretherton], 26 Aug 1984, Folder 80, Earth System Sciences Collection, NCAR, Boulder, CO.

9. Earth System Sciences Overview 86, Draft with Comments, 7 Nov 1985, Folder 37, Earth System Sciences Collection, NCAR, Boulder, CO.

10. Newspaper article by Joan Levine, ‘The View from Out There…’ *The Tribune*, 25 Oct 1983, Folder 101, Earth System Sciences Collection, NCAR, Boulder, CO.

11. Letter from Francis Bretherton to Shelby Tilford, 3 Nov 1986, personal correspondence with Payson Stevens.

12. Telemail from K. Wolfe to L. McCauley, 19 Feb 1986, Folder 38, Earth System Sciences Collection, NCAR, Boulder, CO. Many of the ESSC’s archival documents are early electronic mail messages. Electronic mail was not in widespread popular use in the 1980s. To use it, one either had to have access to an expensive mainframe computer, or one could use a personal computer and simply purchase electronic mail service through a provider, making use of the provider’s access to a mainframe computer. Early users in the 1980s came largely from academia and scientific institutions. The ESSC used the services offered by Telenet, one of the first internet providers to offer an electronic mail service, which the company called ‘telemail’ (Abbate, 1999, pp. 80, 203). All citations for the ESSC’s electronic mail messages refer to them as ‘telemail’.

13. Telemail from LL [McCauley] to Nancy Ann [Brewster], 27 June 1986, Folder 137, Earth System Sciences Collection, NCAR, Boulder, CO.

14. Stevens outlined his approach to science communication in a TED talk: Payson Stevens (1990) TED2 Talk on Global Warming. Available at: https://www.paysonrstevens.com/science-communication/internetwork-media-incinm/ted2-conference/ (accessed 20 January 2021).

15. Robert Redford (1989) Letter from Robert Redford to Payson R. Stevens. Available at: https://www.paysonrstevens.com/wp-content/uploads/2018/05/Redford-Ltr-INI-1989.pdf (accessed 20 January 2021).

16. Payson Stevens (1990) The Interparliamentary Conference on the Global Environment. Available at: https://www.paysonrstevens.com/wp-content/uploads/2018/07/Gore-Interparliamentary-Conference-on-Global-Env-Agenda-April-1990-HiLite.pdf (accessed 31 January 2021).

17. Payson Stevens (2021) Science Communication. Available at: https://www.paysonrstevens.com/science-communication/ (accessed 20 January 2021).

18. Seasat carried five instruments collecting data on variables like sea surface height, wind speed and direction, sea surface temperature and sea ice conditions. If an electrical short out had not prematurely ended the mission, Seasat would have collected tens of thousands of oceanographic measurements (Conway, 2006).

19. There was, interestingly, still a tension in NASA between encouraging data usage and restricting access. Despite its work to promote the use of Landsat data internationally, Neil Maher argues that NASA ultimately maintained control over the Landsat satellite, the ground stations to which the satellite data was downloaded and the data analysis hardware. NASA also authorized which countries could participate and the kinds of research projects that foreign scientists could undertake with NASA funding (Maher, 2019).

20. Landsat 1’s multispectral scanner (MSS) collected radiometric data from the Earth in four spectral bands (in the green, red and two near infrared spectral regions). The MSS received reflected light from the Earth and transferred it, via an oscillating mirror, through filters that selected for the four different wavelength intervals and recorded the data that was then beamed to ground stations on Earth. To create colour images, each spectral band was projected through a particular colour filter, which rendered a ‘false color image’ (Short et al., 1976).

21. Personal correspondence.

22. National Academy of Sciences (2021) IGY ‘Planet Earth’ Posters and Booklet. Available at: http://www.nasonline.org/about-nas/history/archives/milestones-in-NAS-history/international-geophysical-year-files/igy-picture-galleries/planet-earth.html (accessed 31 January 2021).

23. Newspaper article by Joan Levine, ‘The View from Out There…’ *The Tribune*, 25 Oct 1983, Folder 101, Earth System Sciences Collection, NCAR, Boulder, CO.

24. Telemail from P. Stevens to S. Tilford, et al., 3 Jan 1986, Folder 55, Earth System Sciences Collection, NCAR, Boulder, CO.

25. Telemail from P. Stevens to L. McCauley et al., 12 Jun 1986, Folder 50, Earth System Sciences Collection, NCAR, Boulder, CO; Art List, [26 Jun 1986], Folder 50, Earth System Sciences Collection, NCAR, Boulder, CO.

26. Letter from S.G. Tilford to D.G. Rea, 17 Oct 1986, Folder 77, Earth System Sciences Collection, NCAR, Boulder, CO.

27. Note from Laura [Lee McCauley] to Sarah [Hawkins], 16 May 1986, Folder 30, Earth System Sciences Collection, NCAR, Boulder, CO. Stevens can be seen wearing an ESS t-shirt in a picture taken of him and Robert Redford at the Greenhouse Glasnost conference. See: Payson Stevens (2021) Greenhouse Glasnost Conference, Robert Redford’s Sundance, 1989. Available at: https://www.paysonrstevens.com/science-communication/internetwork-media-incinm/redford-sundance-conference/ (accessed 20 January 2021).

28. Recall the iconic ‘Pale Blue Dot’ photograph taken by *Voyager 1* on 14 February 1990 when the spacecraft was 6 million kilometers from Earth. The picture was narrated to great effect by Sagan (1994).

29. Memorandum from Payson Stevens to Shelby Tilford, et al., 27 Mar 1986, Folder 32, Earth System Sciences Collection, NCAR, Boulder, CO; PMS Color Guide (2021) PMS 301 C. Available at: https://www.pmscolorguide.com/coated/pms-301-c (accessed 31 January 2021).

30. Earth System Science Media Support: Preliminary Budgets, [17 Oct 1985], Folder 120, Earth System Sciences Collection, NCAR, Boulder, CO; Memorandum from Payson Stevens to Shelby Tilford, et al., 27 Mar 1986, Folder 32, Earth System Sciences Collection, NCAR, Boulder, CO; Memorandum and Attachments from Laura Lee McCauley to ESSC Members, et al., 15 May 1986, Folder 71, Earth System Sciences Collection, NCAR, Boulder, CO; Memorandum from Payson Stevens to Shelby Tilford, et al., 27 Mar 1986, Folder 41, Earth System Sciences Collection, NCAR, Boulder, CO; Memorandum from Paul Blanchard to Francis [Bretherton] et al., 18 Apr 1986, Folder 118, Earth System Sciences Collection, NCAR, Boulder, CO; Memorandum from Payson Stevens to Paul Blanchard and Laura Lee McCauley, 29 May 1986, Folder 72, Earth System Sciences Collection, NCAR, Boulder, CO; Letter from Francis P. Bretherton to ESSC Members, 6 Apr 1987, Folder 79, Earth System Sciences Collection, NCAR, Boulder, CO; Telemail from J. Eddy to S. Tilford, et al., 14 Mar 1987, Folder 77, Earth System Sciences Collection, NCAR, Boulder, CO.

31. Telemail from P. Stevens to S. Tilford, et al., 3 Jan 1986, Folder 55, Earth System Sciences Collection, NCAR, Boulder, CO.

32. Telemail from P. Stevens to S. Tilford, et al., 3 Jan 1986, Folder 55, Earth System Sciences Collection, NCAR, Boulder, CO.

33. Telemail from N. Brewster to L. McCauley, 12 Jun 1986, Folder 50, Earth System Sciences Collection, NCAR, Boulder, CO.

34. Handwritten notes, [1986], Folder 32, Earth System Sciences Collection, NCAR, Boulder, CO; Handwritten notes, [1986], Folder 136, Earth System Sciences Collection, NCAR, Boulder, CO; Typed notes, [1986], Folder 50, Earth System Sciences Collection, NCAR, Boulder, CO.

35. Telemail from N. Brewster to L. McCauley, 27 Jun 1986, Folder 137, Earth System Sciences Collection, NCAR, Boulder, CO.

36. Payson Stevens (2021) Earth System Science/ESS. Available at: https://www.paysonrstevens.com/science-communication/internetwork-incini/earth-system-science/ (accessed 31 January 2021).

37. Telemail from P. Stevens to L. McCauley et al., 12 Jun 1986, Folder 50, Earth System Sciences Collection, NCAR, Boulder, CO; Art List, [26 Jun 1986], Folder 50, Earth System Sciences Collection, NCAR, Boulder, CO.

38. Telemail from L. McCauley to J. Baker et al., 23 Jun 1986, Folder 137, Earth System Sciences Collection, NCAR, Boulder, CO.

39. [ESSC] Press Conference Invitee Recommendations, [1986], Folder 50, Earth System Sciences Collection, NCAR, Boulder, CO.

40. The ESSC treated ‘human activities’ as external to the Earth system in the sense that, though human activities could affect the Earth system and Earth processes would have an effect on humans, these relationships would require economic, social, and political analyses beyond the scope of the ESSC’s mandate and expertise (NAC, 1988, p. 19).

41. An important historical source for the ESSC’s wiring diagram are the visual depictions of flows of matter and energy that emerged in various Earth science disciplines beginning in the 1940s (Hutchinson, 1948, p. 223, 1970; Taylor & Blum, 1991). Carbon cycle diagrams became increasingly common in the Earth sciences by the 1960s and 1970s, appearing in a variety of publications including textbooks, with later work representing interconnected climate processes with similar kinds of diagrams (Barry & Chorley, 1968, pp. 25–26; United States Committee for the Global Atmospheric Research Program, 1975, pp. 13–14). Though the ESSC called their diagrams ‘wiring diagrams’, the representations have similarities not just with circuit wiring diagrams in use since the nineteenth century but also with flowcharts or flow diagrams that are used to represent structural relationships, algorithmic steps, and/or processes. The earliest flow charts – called ‘process charts’ – were developed by Frank and Lillian Gilbreth in the early 1920s to represent (and hopefully optimize) workplace processes (Gilbreth & Gilbreth, 1921). By 1949, John von Neumann and Herman Goldstine had proposed the use of ‘flow diagrams’ to depict operating instructions for programming computers (Hartree, 1949, p. 112). These were also called wiring diagrams. According to historian Mark Priestley, drawing computer programs as wiring diagrams was a common practice beginning with the programming of ENIAC in the 1940s. Since the ENIAC’s switches and wires required physical configuration before executing operations, its programs were written out as electrical wiring diagrams rather than as lists of commands (Priestley, 2011). This historical legacy of drawing computer programs as flowcharts resembling wiring diagrams continued into the 1980s, at least as a pedagogical tool.

42. In fact, even the establishment of the spatial scale for ESS was problematic. In a systems approach, the system under study needs to be defined. It is not given or final or simply ‘out there’ in an obvious or natural way. Debates arose within the ESSC about where to draw the boundaries of the Earth system, and whether it extended down to the center of the planet and also incorporated the outer reaches of the atmosphere and even the sun. Ultimately, the upper boundary of the Earth system was placed at the mesopause (around 80-90 km above the Earth’s surface). The ESSC also split the Earth system into two timescales (decades to centuries and thousands to millions of years) that had implications for its spatial scale. Only the Earth system on timescales of thousands to millions of years included processes that occurred in the center of the Earth. For timescales of decades to centuries, longer-term processes were considered external to the system (NAC, 1988, p. 91).

43. There were actually three wiring diagrams included in the ESSC’s *Closer view* (1988): A complex and a simplified wiring diagram depicting Earth system processes for timescales of decades to centuries, and a third diagram depicting Earth system processes for timescales of thousands to millions of years. To avoid excessive confusion, this paper ignores the third diagram.

44. While NASA never enacted the ESSC’s specific ESS research program, it did obtain Congressional approval and appropriations in 1989 for its Mission to Planet Earth with its EOS satellite platforms that began flying in the late 1990s, though in admittedly diminished forms. In 1999, Terra became the first EOS satellite to launch, with instruments collecting data on the Earth’s clouds, ice, water, land surfaces, and radiation budget, as well as atmospheric carbon monoxide. NASA’s EOS satellites, along with those from other space-faring countries, continue to contribute to the construction of global datasets for Earth and environmental science research, thus facilitating the study of the planet as an interconnected system (Conway, 2008, 2014; Goldstein, 2007; Lambright, 1994a, 1994b; Leshner, 2007; Leshner & Hogan, 2019).

45. Humanities scholarship on the Anthropocene is vast. Bonneuil and Fressoz (2015) and Dryzek and Pickering (2019) provide excellent overviews.

46. Before 1986, the ‘Earth system’ referred almost exclusively to an electrical grounding system or a composting toilet.

47. These search results were obtained on 27 January 2021 from the *Science* databases at JStor (for 1880-2014) and the *Science* website (for 2015-present), and *Nature’s* online journal archive (1869-present).

48. NASA Global Climate Change (2021) The study of the Earth as an integrated system. Available at: https://climate.nasa.gov/nasa_science/science/ (accessed 31 January 2021); NASA’s Earth Observing System (2021) Missions: Earth System Science Pathfinder Program. Available at: https://eospso.nasa.gov/mission-category/11 (accessed 31 January 2021).

49. Google’s ngram viewer searches for words or phrases within a ‘corpus of books’ (e.g. ‘British English’, ‘English Fiction’, ‘French’) over a selected number of years and produces a graph which plots the trend in use of the particular word/phrase compared to the entirety of the corpus. See: Google Books (2021) Google Books Ngram Viewer. Available at: https://books.google.com/ngrams/info (accessed 31 January 2021).
